# The Emergence of Genome Editing—Innovation Network Dynamics of Academic Publications, Patents, and Business Activities

**DOI:** 10.3389/fbioe.2022.868736

**Published:** 2022-04-14

**Authors:** Natalie Laibach, Stefanie Bröring

**Affiliations:** ^1^ Laboratory for Sterol and Terpenoid Metabolism in Plant Development and Stress Responses, Department of Plant Synthetic Biology and Metabolic Engineering, Centre for Research in Agricultural Genomics (CRAG), Barcelona, Spain; ^2^ Chair Entrepreneurship and Innovative Business Models, Center for Entrepreneurship, Innovation and Transformation, Ruhr-University Bochum, Bochum, Germany

**Keywords:** genome editing, social network analyses (SNA), innovation, technology and innovation management, sustainability, biotechnology

## Abstract

Transformative societal change can both be triggered and influenced by both macro-level political means and the emergence of technologies. Key enabling technologies and therein biotechnology hold the power to drive those changes forward, evolving from breakthrough academic discoveries into business activities. Due to its increasing empirical relevance, we picked genome editing as an example for an emerging technology and extracted publication, patent, and company data from the years 2000 to 2020. By drawing upon social network analysis, we identify major networks and clusters that are dominating the respective time and layer. Based on these networks, we draw vertical connections between scientific knowledge, patented technologies, and business activities to visualize the interlevel relationships between actors through technological development. Thereby, we identify network dynamics of the emergence of genome editing, the most important actors and clusters evolving, and its spread into different areas.

## 1 Introduction

Amid fossil resource and land scarcity, climate change, and a growing and aging world population, sustainable and resource-efficient strategies are required to enable the prosperity of future generations. To comply with the United Nations’ Sustainable Development Goals (SDGs), which address among others climate change, health, innovation, and sustainable consumption, societal change and sector-spanning activities are needed that are, mutually, driven by and fostering new technological developments (United Nations, 2015; El-Chichakli et al., 2016). If they hold great potential, niche innovations manifesting in start-ups can become key enabling technologies (KETs) (Geels and Schot, 2010; Hausknost et al., 2017; Priefer et al., 2017; Philp, 2018)—and industrial biotechnology is among them (Dupont-Inglis and Borg, 2018; Małyska and Jacobi, 2018): “Industrial biotechnology is broadly accepted as one of the EU’s core technological strengths and has been recognized as a ‘key enabling technology’ which can help enable a more competitive and sustainable bioeconomy” (Dupont-Inglis and Borg, 2018, p. 141).

Certainly, one of the most critical developments in recent years peaking in the reception of the Nobel Prize (Cohen, 2020; Strzyz, 2020) is genome editing or CRISPR/Cas9 (clustered regularly interspaced short palindromic repeats/CRISPR-associated protein 9)—a tool to induce precise modifications in the genome of any organism. This reveals great opportunities not only in plant breeding but also for new medicinal applications. Genome editing such as CRISPR/Cas9 and its fewer famous predecessors such as zinc finger nucleases (ZFN) and transcription activator–like effector nucleases (TALENs) have the potential to (and currently do) improve diverse biotechnological applications to promote the transition toward a bio-based economy (Barrangou and Doudna, 2016; Laibach et al., 2019). It could alter crops with respect to climate change adaptation (e.g., drought resistance), thus ensuring food security, reducing the amount of fertilizer and pesticides, which will benefit the environment and could empower farmers in developing countries (Lopes, 2015; Huang et al., 2016; Yang et al., 2017; Goold et al., 2018). Moreover, the plants and microorganisms could be designed to provide optimal feedstock for non-food products and applications (Lopes, 2015; Nemhauser and Torii, 2016; Flores Bueso and Tangney, 2017), and medical treatments can be developed (Barrangou and Doudna, 2016) by means of this novel approach.

Given its impact and importance, genome editing is an outstanding case from a technology and innovation management point of view that is disrupting its own technological regime (Martin et al., 2020). Here, genome editing can be defined as an emerging technology as it fulfills the five criteria radical novelty, relatively fast growth, coherence, prominent impact, and uncertainty and ambiguity developed by Rotolo et al. (2015); Rotolo et al., 2015. Patent analysis of specific application areas points toward future increase in technological development (Gupta et al., 2020) and already identified some key players connected to the patent licensing of major scientists involved, including the recent Nobel laureates (Egelie et al., 2016). However, a systemic change is only possible if new potential technologies diffuse from scientific knowledge into the market applications and efficient transfer systems are established (Geels and Schot, 2010; Vandermeulen et al., 2012; Graff and Sherkow, 2020). Especially when emerging technologies have the potential to change different areas such as agriculture and medicine, the dynamics of the innovation networks from science to business can reach a high level of complexity (Golembiewski et al., 2015a). The networks or ecosystems of involved actors can illustrate the most important players in this field and visualize key connections between scientific research and business application. This appears to be very important because although it is possible to carry out scientific research, technological developments, and their commercialization by oneself (single university, research institute, and company), it is assumed that precisely collaboration and networking at academic and technological levels create the needed synergic effect, and thus the partners may mutually develop faster, achieve better results, and innovate more effectively (Daugherty et al., 2006; Hewitt-Dundas, 2006).

As genome editing techniques and its development have been extensively reviewed, we aimed to shed light on its development from a technology and innovation management perspective. Drawing on the theoretical background of emerging technologies in innovation networks and ecosystems (Markard and Truffer, 2008; Rotolo et al., 2015; Campbell and Carayannis, 2016), we assume that over time, network clusters surrounding the most successful technological applications emerge and move from science to the business layer. By mapping these clusters, we can provide empirical evidence for the advancement of genome editing whereby policymakers and managers can identify the collaboration partners, emerging/retarding, and area potential setscrews to intervene.

## 2 Technological Innovations Are Embedded in the Context of Their Ecosystems

### 2.1 Innovation Ecosystems and Networks of Emerging Technologies

Innovation ecosystems generate economic dynamics of different actors (universities, research organizations, and enterprises) and in space, which facilitate technological development, through their human capital, knowledge, and material resource synergy (Majava et al., 2013; Binz et al., 2014; Markard et al., 2015). Hence, technology transfer through interconnections and synergy of scientific institutes, technological projects, and established firms and technology-based ventures/start-ups are considered to be highly important for dynamic technological systems (Bergek et al., 2008, 2015; Tigabu et al., 2015). Firms engaged in innovation ecosystems are also known to obtain a higher total value than without cooperation (Hellström et al., 2015; Xu et al., 2018). These emerging networks within innovation ecosystems can be used for a better understanding of such systems and their drivers and studies using social network analysis (Xu et al., 2018). This has been used to identify dynamics, key actors, and areas that facilitate the market implementation of scientific developments using publication data (Kajikawa et al., 2007; de Souza et al., 2015), combining patents and publications (Curran et al., 2010; Binz et al., 2014; Goeldner et al., 2015; Gaviria and Kilic, 2021), and including company information (Xu et al., 2018). The key actors and their roles can be identified by their network values (Stolz and Schlereth, 2021). The cooperation and diffusion of knowledge between universities and companies and across disciplines is enhanced by collaborative research activities and institutionalized infrastructures, thus providing opportunities for academic entrepreneurship (Etzkowitz, 2012; Wu et al., 2018).

### 2.2 Social Network Analysis to Identify the Innovation Ecosystem of Genome Editing

One major aim of this study is to investigate the scientific and technological layers within the genome editing ecosystem framework so as to entangle the technologies developing clusters and growth pathways toward its market commercialization. Therefore, we analyze the publication, patent, and company database landscapes, which is one widely used approach in scientific publications to investigate and understand the emerging technologies and products (Dai et al., 2015; Goeldner et al., 2015) and, in particular, novel technologies in the context of bio-economy (Milanez et al., 2014; Berg et al., 2018). Real market applications of scientific knowledge and intellectual property are beneficial in terms of revenue generation, general business development, and novel goods and service market extension (Siegel et al., 2007). Hence, to identify the important actors and technology lines in genome editing, we conducted a research strategy that first identifies the major actors and areas in genome editing using social network analysis and thus quantifies the conjuncture of the industry. Afterward, we linked the three layers of publication, patent, and company networks and highlighted the corresponding paths.

#### 2.2.1 Data Generation

To obtain the relevant datasets for our analysis, we extracted publication data from Web of Science (WoS; https://apps.webofknowledge.com/), which is one of the most popular and comprehensive existing scientific search engines that has already been used for bioeconomic research description and also to describe the rise of genome editing research in recent years (Mao et al., 2015; Eriksson et al., 2018). This resulted in 9,992 publications from which bibliographic information was extracted. Publication analysis already was applied to describe the scientific ecosystems using bibliometrics, which is an important instrument for analysis and evaluation of scientific research output (Van Raan, 2005; Cobo et al., 2015; Martínez et al., 2015). To gain insights into technological dynamics in a certain area, patent information is widely used as it is relevant for strategic planning and to evaluate R&D activities (Ernst, 2003; Chen et al., 2013). The patent data for the research were obtained from Derwent Innovations Index (DII; www.derwentinnovation.com/)—one of the most comprehensive existing patent information databases, which was used as a reliable source for conducting similar research studies (Ma and Porter, 2015; Berg et al., 2018) and resulted in 27,583 patent families. The corresponding search strings can be found in Supplementary Table S1, Supplementary Material. The publications were restricted to English articles, and in addition, the timespan from 2000 to 2020 was applied in order to incorporate the beginning of genome editing. Data for the business layer data, in total 606 hits, were obtained using the Pitchbook (https://pitchbook.com//) database. Here, it was necessary to change the search string because of the peculiarities due to which the Boolean operators and wildcards cannot be used. Company foundations and business deals were collected and used for further analysis.

#### 2.2.2 Horizontal Analysis

Social network analysis (SNA) is recognized as a useful tool for the description of central actors, hubs, and main movements of certain fields (Sternitzke et al., 2008; de Souza et al., 2015) and allows for a comprehensive visualized understanding of the flows between the top-actors of a certain technology field (or other). SNA possesses a range of analysis and visualization methods, for example, building collaboration and citation networks (Perianes-Rodríguez et al., 2010). All calculations, visualization, and coefficients for social networks were generated similarly for scientific, technology, and business layers as follows. The necessary organizational data were collected from the corresponding database (WoS, DII, and CrunchBase) and consolidated by periods (2000–2009, 2010–2014, 2015–2017, and 2018–2020). Then, using Microsoft Excel, datasets were redistributed in order to sort all authors of each particular entry. Then, the organization of one entry—that is, affiliations of publication authors, non-persona co-assignees—were paired among each other manually (e.g., if the document consists of the authors from MIT, Harvard University, and Broad Institute, there are next links: MIT—Harvard University, MIT—Broad Institute, Harvard University—Broad Institute; in case of the same institution per entry, self-nominating organizations were eliminated). For the business layer, company connections were obtained by merger and acquisition activities and co-founders who are listed in different companies. The network as such includes several indicators (degree centrality, closeness centrality, betweenness centrality, network density, average path length, and clustering coefficient) which were calculated using the software R 3.5.3 and the RStudio desktop version 1.1.463. Specifically, we applied the packages *igraph* (Csardi and Nepusz, 2006), *ggplot2* (Wickham, 2016), and *readr* (Wickham et al., 2017). The full script can be found in Supplementary Material S1.1. A pajek file was then created to load the data into Gephi (Bastian et al., 2009) so as to obtain standardized visualization of the network. The Fruchterman–Reingold and expansion algorithm were used, and the network parameters of adapted networks were calculated in Gephi.

#### 2.2.3 Vertical Analysis

The bridging of the three analyzed layers was conducted by matching organizations that occurred both in publication and patent or patent or company data. To visualize the most important clusters among each layer, we further considered the 100 actors with the highest degree of centrality among the years and indicated the organization’s technological area, if applicable (universities for instance are usually multidisciplinary). In addition, we included all those organizations that have connections throughout the layers.

## 3 Genome Editing Spans Different Sectors and Connects Interdisciplinary Actors

**FIGURE 1:**
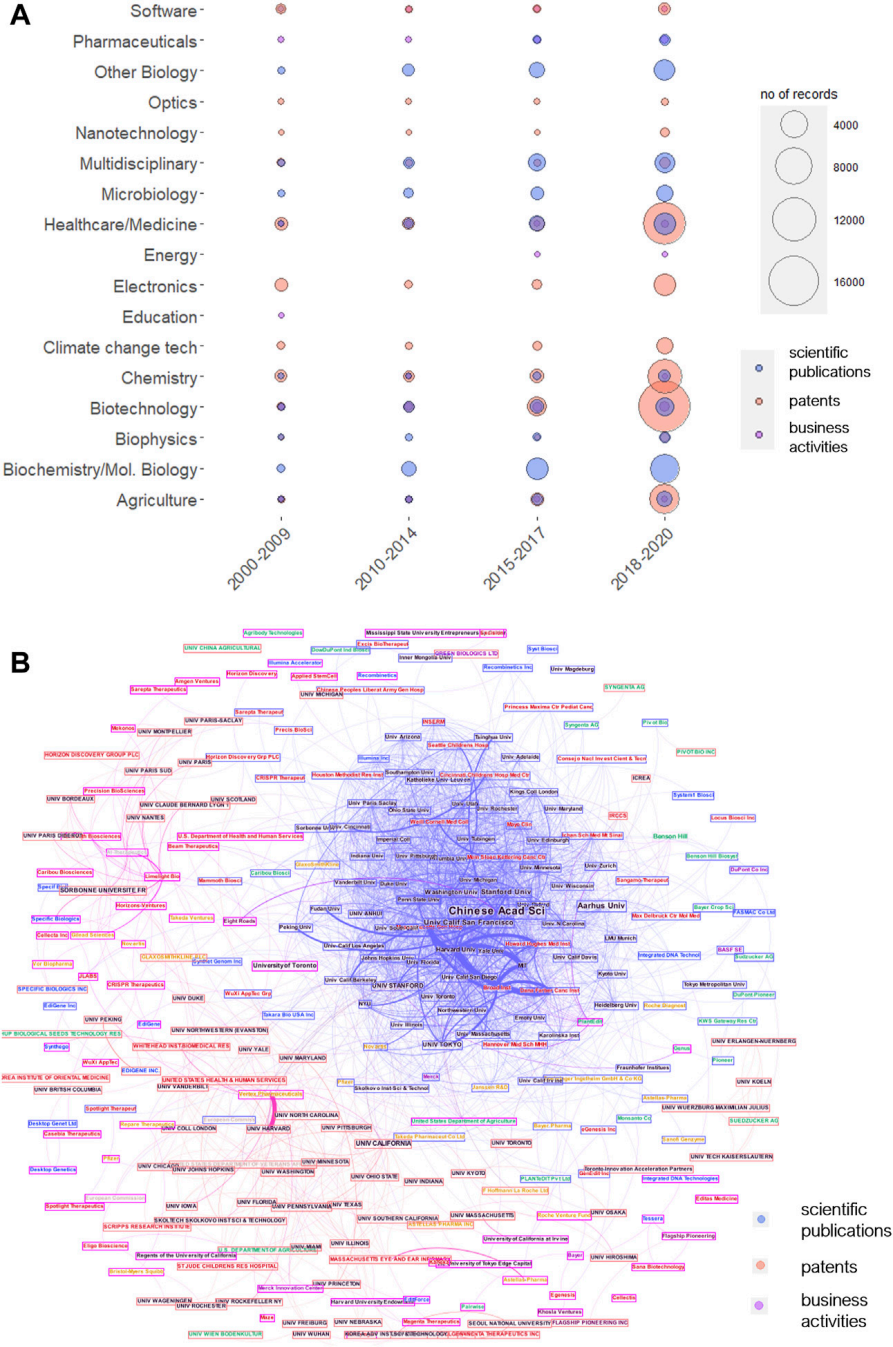
**(A)** Thematic areas of genome editing by record number during the periods of 2000–2009, 2010–2014, 2015–2017, and 2018–2020. The data shown display the 15 areas with the highest record number per period. Data for academic publications (s), patents (p), and company data (c) were obtained using Web of Science, DII, and Pitchbook **(B)** Network of institutions active in academic publications, patents, and business from 2018 to 2020. Network generated from publication (s, WoS), patent (p, DII), and company (c, Pitchbook) data from 2018 to 2020, using the institutions with the top 100°, visualized by Gephi. Thematic areas, red; medicine, orange; pharmaceuticals, pink; chemistry, green; agriculture, blue; biotechnology, dark red; venture capital, gray: other.

To explore networks emerging around genome editing, we first obtained publication, patent, and company data for genome editing in the period from 2000 to 2020 and retrieved the dominant actors and second identified the most dominant areas (Figure 1A). Each information is then at last used to conduct SNA to delineate network positions and collaborations. The number of entries in each dataset rapidly increased over the last 5 years (Supplementary Figure S1), reflecting the success of the CRISPR/Cas9 method from 2012 onward (van Erp et al., 2015; Barrangou and Doudna, 2016). Before, TALEN and other genome editing approaches were identified and patented, but none of these resulted in a comparable boost in patenting and publication activities. The major actors in terms of the record numbers of academic publications and patents are Harvard University with its associated institutes (MIT and Broad Institute), the University of California, the Chinese Academy of Science, and the French research institutes CNRS and INSERM (Supplementary Figures S2, S3). The most active companies in terms of patenting are Monsanto (now Bayer) and PioneerHiBred from the agricultural sector, which also displays the most relevant areas for CRISPR/Cas9 application (Supplementary Figures S2–S4).

We then performed social network analysis to identify the most relevant clusters and leading organizations in each period of analysis. The networks for each layer reflected the development of genome editing throughout the years until a saturation was attained after 2015 for patents and academic publications, making the networks nearly impossible to read (Supplementary Figures S6–S9). However, some companies such as Monsanto that hold many patents but were scarcely engaged in collaboration largely disappear from the collaborative landscapes.

The big clusters are centered around French (i.e., Institute Pasteur, CNRS, INSERM), Chinese (i.e., Chines Academy of Science), and two U.S. institutions from Massachusetts (i.e., Harvard University, MIT) and California (i.e., University of California). In the later years, the companies emerged and formed these innovation ecosystems, many specializing in genome editing, that is, Editas Medicine, CRISPR Therapeutics, and Mammoth Biosciences. To find not only the actors with the most ties but also such that are important bridges for technology transfer and collaboration, we calculated the degree of centrality and the betweenness centrality of the networks. While the former displays the number of ties, the latter is considered an indicator for power within the network (Burt, 2003; Stolz and Schlereth, 2021). From 2000 to 2009, Sangamo Therapeutics (Sangamo Biosciences) and Iowa State University have the highest values, reflecting an important tying position in the early genome editing technology development before CRISPR/Cas9 (Supplementary Figure S5). While Sangamo Therapeutics can hold its position as a very collaborative institution, especially in academic publications, the organization with the most records is gaining network power.

**FIGURE 2:**
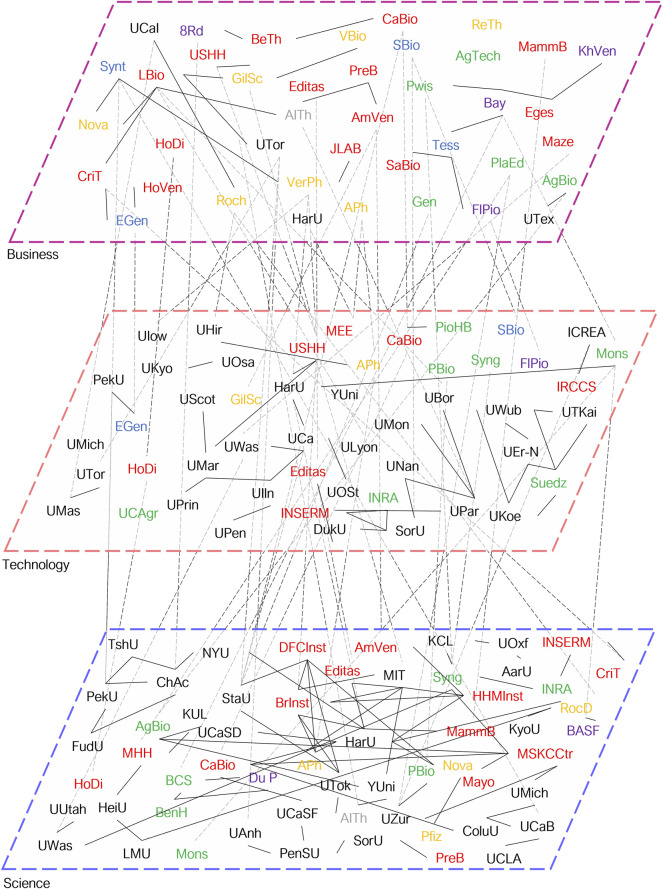
Reduced network of institutions active in academic publications, patents, and business from 2018 to 2020. Connections are based on a network generated from publication (Science, WoS), patent (Technology, DII), and company (Business, Pitchbook) data from 2018 to 2020, using the institutions with the highest degrees and betweenness centralities, calculated by Gephi. Thematic areas, red; medicine, orange; pharmaceuticals, pink; chemistry, green; agriculture, blue; biotechnology, dark red; venture capital, gray: other. Abbreviations see Supplementary Table S2.

But which networks and actors are currently active in which areas and around which areas do the clusters evolve? To show the actual state of institutions involved in genome editing, we combined the data from academic publications, patents, and business activities in single network analysis in the period from 2018 to 2020 and included, where possible the thematic area. Figure 1B and Figure 2 show the whole network of organizations truncated by the degree of centrality. The major clusters in agricultural sciences involve the U.S. Department of Agriculture, the University of Cologne, Benson Hill, and others. Medical clusters are dominated by U.S. universities and the U.S. Health and Human Services Department and encompass not only medical but also most pharmaceutical companies such as Novartis or Pfizer. The cluster with a majorly pharmaceutical focus involves CRISPR/Cas9 start-ups with a medical focus and mostly French universities. Moreover, some companies from two different areas begin to collaborate. For instance, DowAgrosciences, a big agricultural player, is co-assigning inventions in genome editing with Sangamo Therapeutics from the medical biotechnology area. The latter is also involved in business-academic networks, for example, with the University of California. Another example is Syngenta which is scientifically collaborating with the Memorial Sloan Kettering Cancer Center (see also Figure 2). This again highlights the boundary-spanning potential of genome editing that as a KET triggers cross-sectoral innovation and possibly convergence, thus giving rise to new innovative systems.

## 4 Discussion

The emergence of genome editing, especially after discovering the CRISPR/Cas9 methodology, resulted in very complex horizontal networks in academia and patents—that reflect also the great diversity of technology transfer models in this technological system (Graff and Sherkow, 2020). Therapeutics and medical solutions are currently the most relevant field for genome editing applications, as our analysis of the patent data and the major company clusters show. The second counts plant science (agricultural biotechnology); however, that is mostly in the hands of established companies such as Bayer/Monsanto, DowDuPont, or Syngenta The discouraging politics in Europe may repel new company foundations and investments (Huang et al., 2016) as is the general ethical discussion around bioengineering for food production (Gupta et al., 2020), which could be the reason behind the observed lack of network density and could be pointing toward uncertainty—and the still emerging character of this technology (Rotolo et al., 2015, 2017). In the same vein, expectations for application in agriculture are remaining high (Maximiano et al., 2021; Hüdig et al., 2022), and in-detail patent analysis prospects a strong increase in product development (Gupta et al., 2020). A most surprising result was that although CRISPR/Cas9 genuinely belongs to microbiology, this area is largely underrepresented in our analysis, although there are many opportunities (van Doren et al., 2013; Barrangou and Doudna, 2016). A possible explanation might be the immediate monetary benefit from the other areas and the comparably early stage of development of the promising field of synthetic biology. As this technology field is also rising, we may see similar developments in near future (van Doren et al., 2013). This could be an asset for a sustainable bioeconomy and a fundus for many new applications to produce high-value chemical compounds etc. (Lopes, 2015; Flores Bueso and Tangney, 2017). Knowledge-intensive fields such as genome editing and biotechnology require a broad and strong boundary spanning (interdisciplinary) scientific base; hence, companies will engage in joint R&D efforts with academia (Akbar et al., 2012; Golembiewski et al., 2015b; Lokko et al., 2018). The development of the CRISPR/Cas9 technology clearly benefited from the engagement of academic institutions and individuals in the commercialization of their ideas, as was shown in other areas before (Etzkowitz, 2012; Wu et al., 2018)—and is likely to be shaped by these in the future (Graff and Sherkow, 2020; Whelan et al., 2020). This is visible in our analysis of public–private networks throughout the layers. The visualization of the technological orientation of each connection might be interesting to detect new emerging fields of science and hence for future research avenues. Moreover, as the data obtained in the present analysis also provide information about authorship (inventor, founder) and geographical location, this can be used to extend the three-layer model upon the spatial perspective and additional connections (Markard and Truffer, 2008).

Although this research is limited by the use of the datasets, it can highlight successful pathways of emerging technologies with an innovation ecosystem. From here, scientists, managers, and policymakers can dig deeper and identify reasons for the success or failure of inventions. Hot spots of technological development, key partners, and application areas can be identified. CRISPR/Cas9 as the major genome editing technology is a vertical technology, spanning different area, which are expanding from medicine and healthcare to agriculture. In addition, the stagnation of successful technology areas into the science or technological layer may be caused not only by the feasibility to design prototypes on an industrial scale but also by political or societal circumstances. Policy-based regulations or public concerns may form a barrier hampering the market entrance and thus the further development of technological clusters. Future research could aim at studying the social legitimacy of major clusters and elaborate on the business models employed by the start-ups centered on CRISPR/Cas9. Overall, our research can help visualize those developments or locked-in states and point toward further necessary actions.

## Data Availability

The data analyzed in this study is subject to the following licenses/restrictions: The patent and company data were purchased from databases provided by Web of Science (WoS; https://apps.webofknowledge.com/), Derwent Innovations Index (DII; www.derwentinnovation.com/), and Pitchbook (https://pitchbook.com//), which are commercial entities. The requests to access these datasets should be directed to NL, natalie.laibach@cragenomica.es.
