# Supplementary material for: The Emergence of Genome Editing—Innovation Network Dynamics of Academic Publications, Patents, and Business Activities
Source: Front Bioeng Biotechnol. 2022 Apr 14;10:868736. doi: 10.3389/fbioe.2022.868736 (PMC9049213; doi:10.3389/fbioe.2022.868736)
Supplement: Supplementary file 1 [file DataSheet1.DOCX]

Supplementary Material

# Supplementary Methods

**Supplementary Table S1:** Databases and corresponding search strings for data retrieval

| **Data & Database** | **Search string** | **Number of results** |
| --- | --- | --- |
| Academic publications: Web of Science | (TS=("genetic editing" OR "gene scissor" OR "gene editing" OR "genome editing" OR "genome edit" OR "ZFNs" OR "zinc finger nucleases" OR "zink-finger nucleases" OR "Crispr/Cas" OR "CRISPR/Cas9" OR "Crispr" OR "transcription activator-like effector nucleases" OR "TALEN")) AND LANGUAGE: (English)  Refined by: [excluding] DOCUMENT TYPES: ( BOOK CHAPTER OR BOOK REVIEW OR BIBLIOGRAPHY OR REVIEW OR LETTER OR DATA PAPER OR BIOGRAPHICAL ITEM OR MEETING ABSTRACT OR CORRECTION OR RETRACTED PUBLICATION OR DATABASE REVIEW OR EDITORIAL MATERIAL OR EARLY ACCESS OR RETRACTION OR REPRINT OR NEWS ITEM OR PROCEEDINGS PAPER ) AND PUBLICATION YEARS: ( 2009 OR 2004 OR 2008 OR 2003 OR 2007 OR 2002 OR 2006 OR 2001 OR 2005 OR 2000 )  Timespan: 2000-2020. Indexes: SCI-EXPANDED, SSCI, A&HCI, ESCI. | **9992**  Date of retrieval: February 25^th^ 2021 |
| **Patents:** | ALL=("genetic editing" OR "gene scissor" OR "gene editing" OR "genome editing" OR "genome edit" OR "ZFNs" OR "zinc finger nucleases" OR "zink-finger nucleases" OR "Crispr/Cas" OR "CRISPR/Cas9" OR "Crispr" OR "transcription activator-like effector nucleases" OR "TALEN") AND DP>=(20000101) and DP<=(20201231); | **27583**  Date of retrieval: July 7^th^ 2021 |
| **Company Data** | Deal Date: From: 01-Jan-2000; To: 01-Jan-2021; Deal Option: Search on a full transaction; Keywords: crispr*; genetic editing*; gene scissor*; gene editing*; genome editing*; genome edit*; zfns*; zinc finger nucleases*; zink-finger nucleases*; crispr/cas*; CRISPR/Cas9*; transcription activator-like effector nucleases*; talen; Include Active Positions; | **606**  Date of retrieval: March 11th 2021 |

## Social network analysis script

library (igraph)

library (readr)

########################################

# Collaboration Network DATA #

########################################

#read and import the csv network file

X <- read.csv("xx.csv", sep=";", header=FALSE)

X <- subset(X, select=c("V1", "V2"))

#Creating a Graph Obeject for Subsequent Analyses

author_graph=graph.data.frame(X, directed=F)

V(author_graph)$size <- degree(author_graph, mode = "all")/5

V(author_graph)$id = V(author_graph)$name

edge_attr(listing_Graph)$weight

degree(author_graph) # number outgoing edges

graph.strength(author_graph) # weighted degree

degree_centr <- centr_degree(author_graph, mode = "all", loops = F, normalized = F)

# betweennes centrality

betw_centr <- centr_betw(author_graph, directed = F, normalized = F)

betweenness(author_graph, v = V(author_graph), directed = F, weights = NULL)

# eigenvector centrality

eig_cent <- eigen_centrality(author_graph, directed = FALSE)$vector

#write density and graph parameters - the directory has to be changed according to your preferences

write.csv(degree_centr, "degree_centrX.csv")

write.csv(betw_centr, "betw_centrX.csv")

write.csv(eig_cent, "eig_centX.csv")

#write pajek file - this can be opened with Vosviewer or Gephi

write.graph(author_graph, "X.net", format = c("pajek"))

# Supplementary Figures


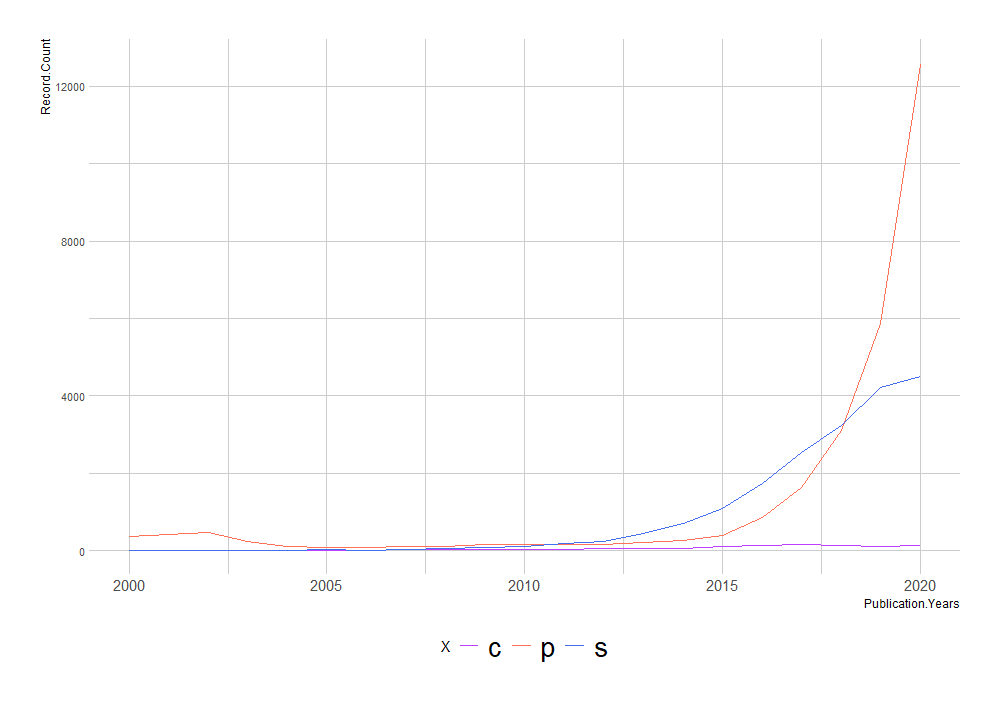


**Supplementary Figure 1**: Number of database entries per year for academic publications (s) , patents (p) and business entries (c).


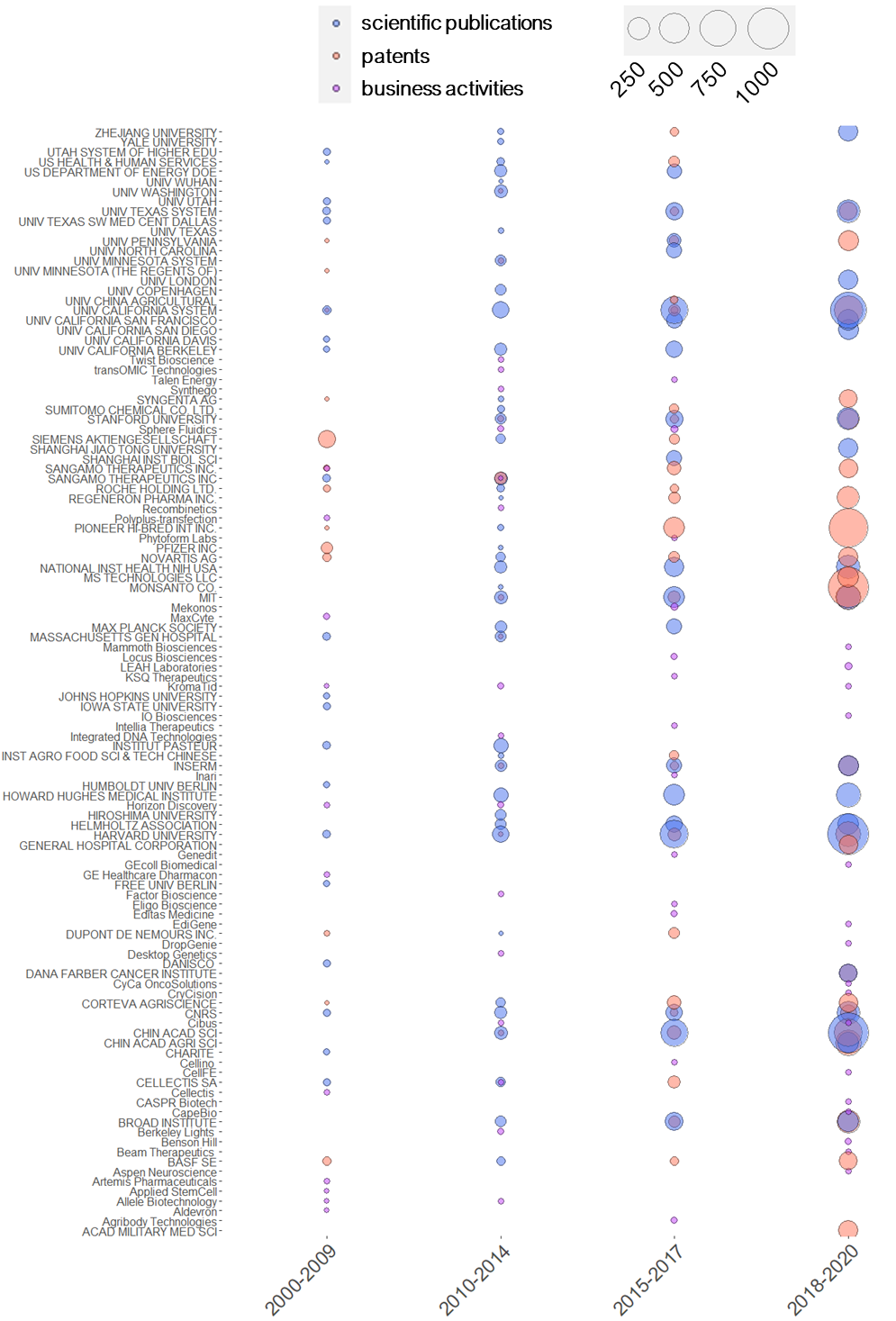


**Supplementary Figure 2** Institutions listed as affiliations, assignees, or company names by record number in the periods of 2000-2009, 2010-2014, 2015-2017 and 2018-2020. The data shown displays institutions among the 20 with the highest record number per period. Data for academic publications (s), patents (p) and company data (c) were obtained using WebofScience, DII and Pitchbook.


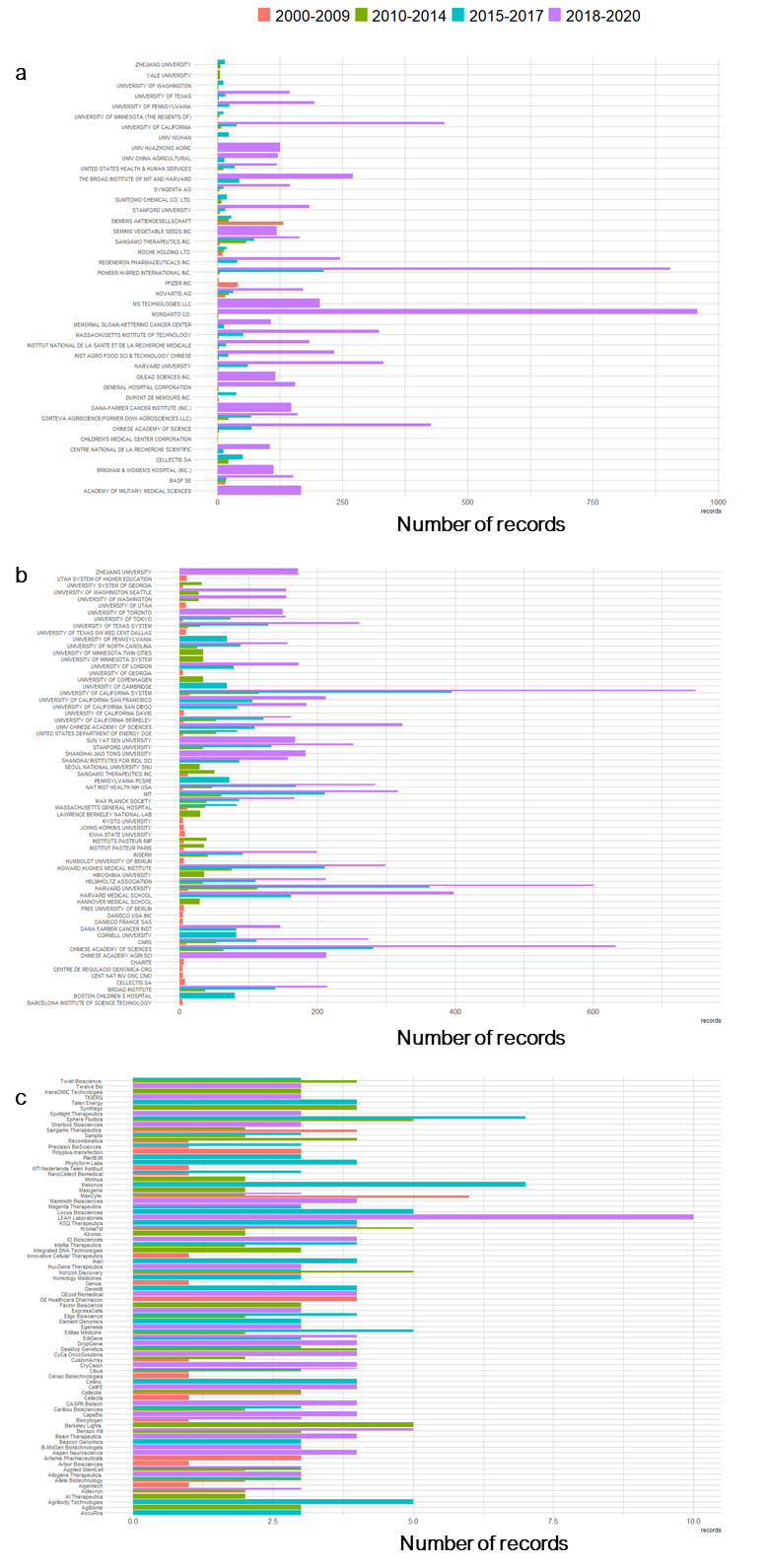


**Supplementary Figure 3** Institutions listed as affiliations, assignees, or company names by record number in the periods of 2000-2009, 2010-2014, 2015-2017 and 2018-2020. The data shown displays institutions among the 30 with the highest record number per period. Data for academic publications (s), patents (p) and company data (c) were obtained using WebofScience, DII and Pitchbook.


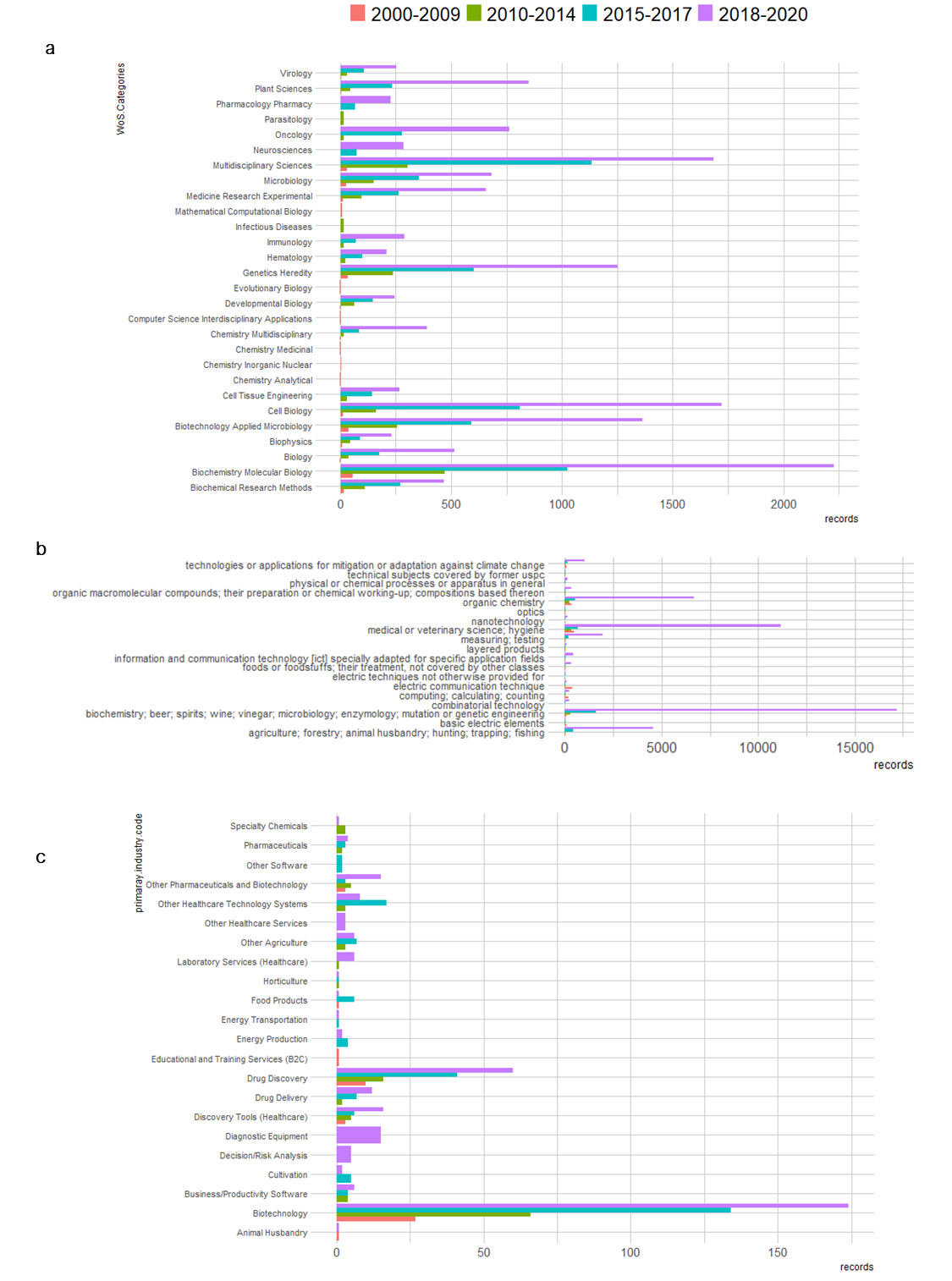


**Supplementary Figure 4** Thematic areas of genome editing by record number in the periods of 2000-2009, 2010-2014, 2015-2017 and 2018-2020. The data shown displays institutions among the 20 with the highest record number per period and display WoS Categories (a), CPC Classes (b) and primary industry codes (c). Data for academic publications (s), patents (p) and company data (c) were obtained using WebofScience, DII and Pitchbook.


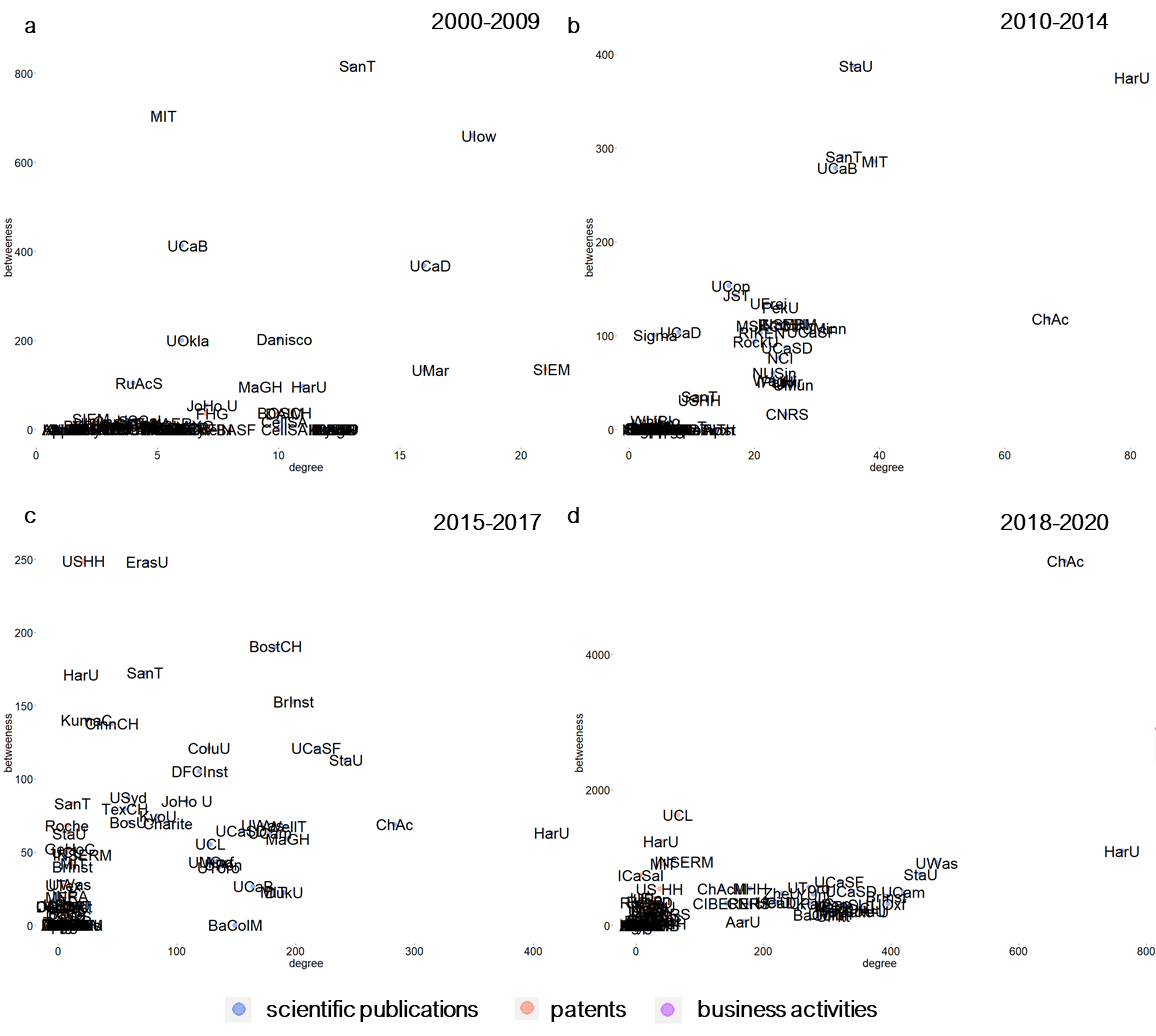


**Supplementary Figure 5** Degree of centrality and betweenness centrality of top 20 institutions in periods of 2000-2009, 2010-2014, 2015-2017 and 2018-2020. The data shown displays institutions among the 20 with the highest degree/betweenness centrality per period. Data for academic publications (s), patents (p) and company data (c) were obtained using WebofScience, DII and Pitchbook. Abbreviations see Supplementary Table 1.


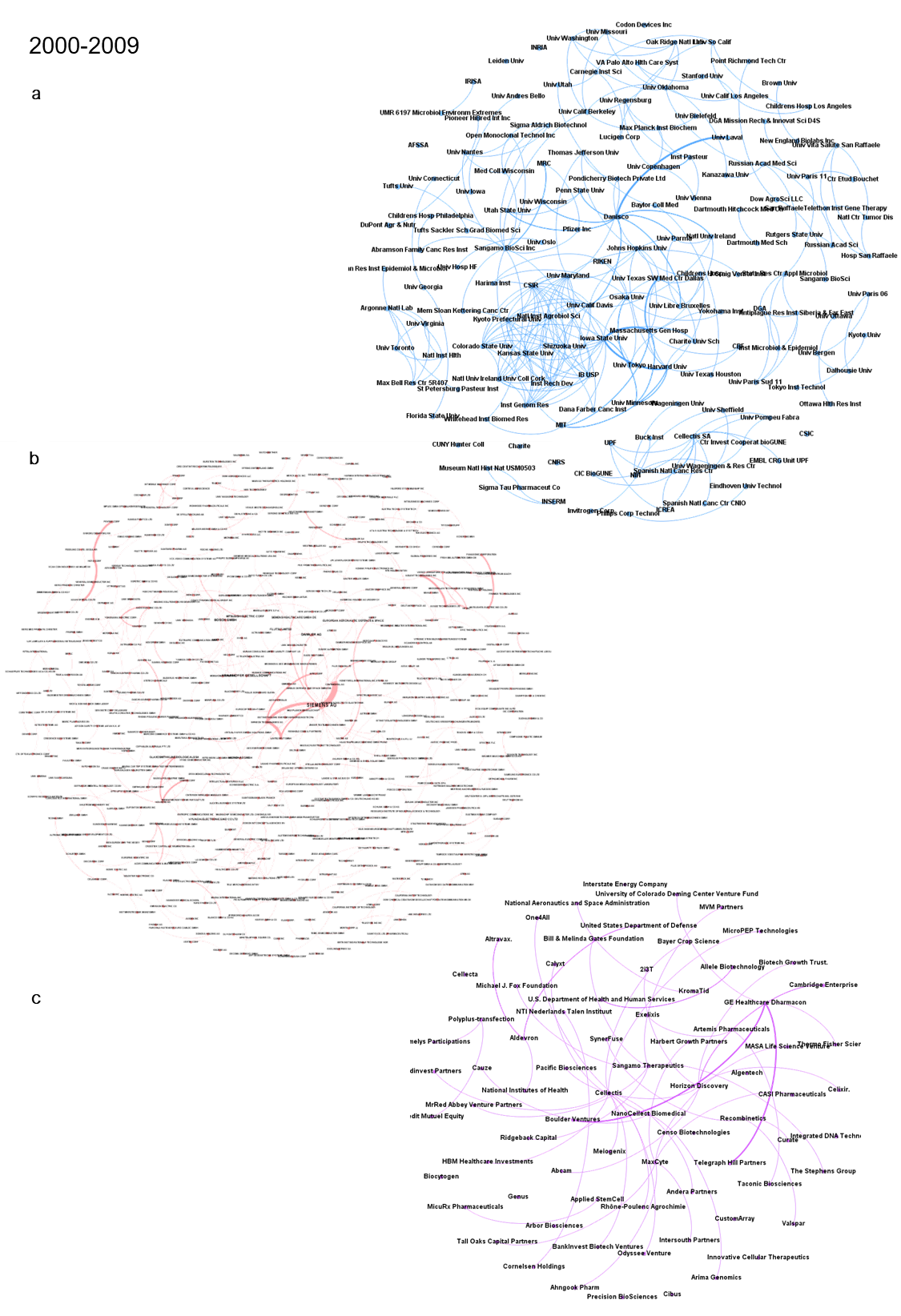
**Supplementary Figure 6:** Networks generated from a publication (WoS) c patent (DII) and c company (Pitchbook) data, in the period from 2000-2009, visualized by Gephi.


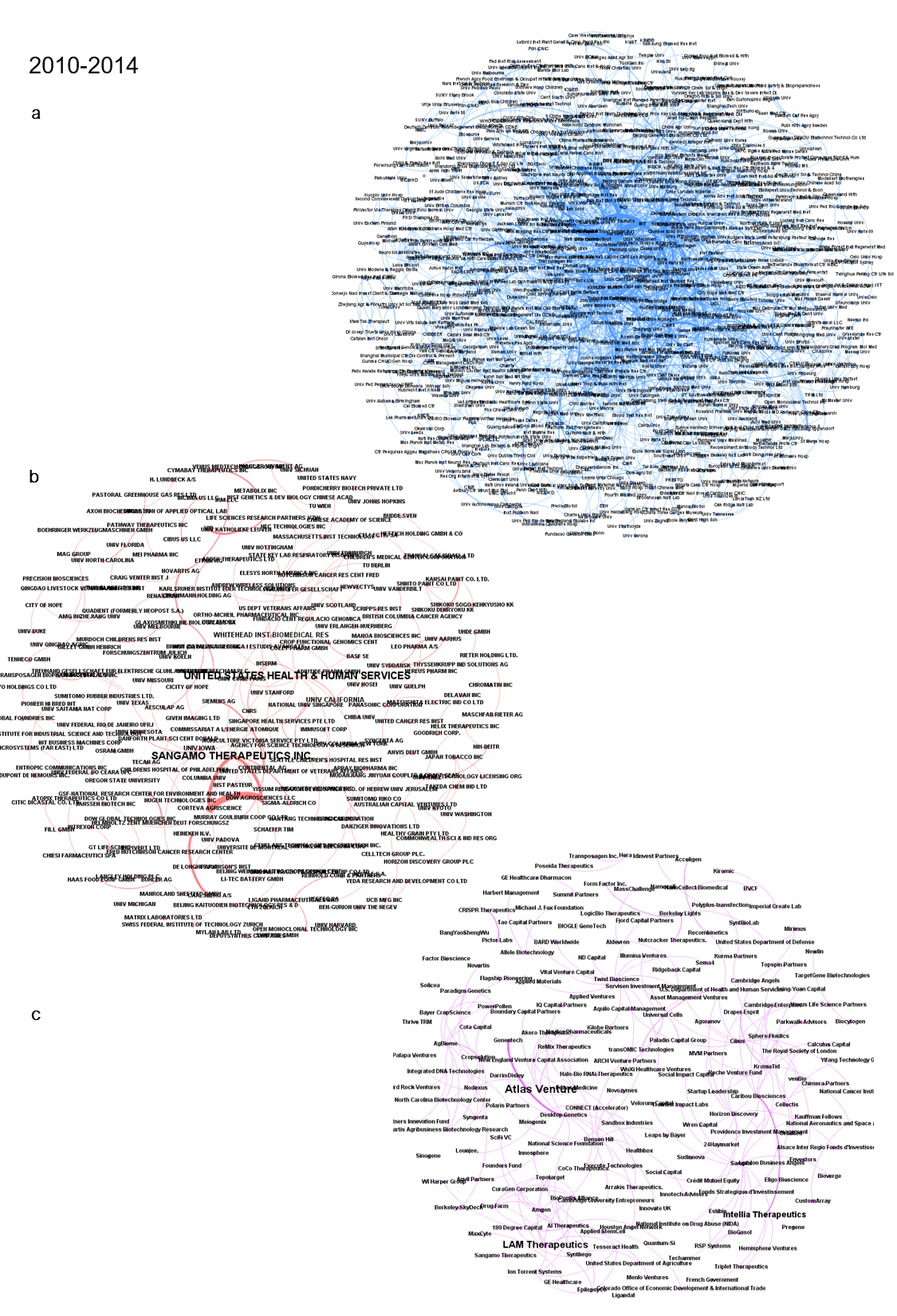


**Supplementary Figure 7:** Networks generated from a publication (WoS) c patent (DII) and c company (Pitchbook) data, in the period from 2010-2014, visualized by Gephi.


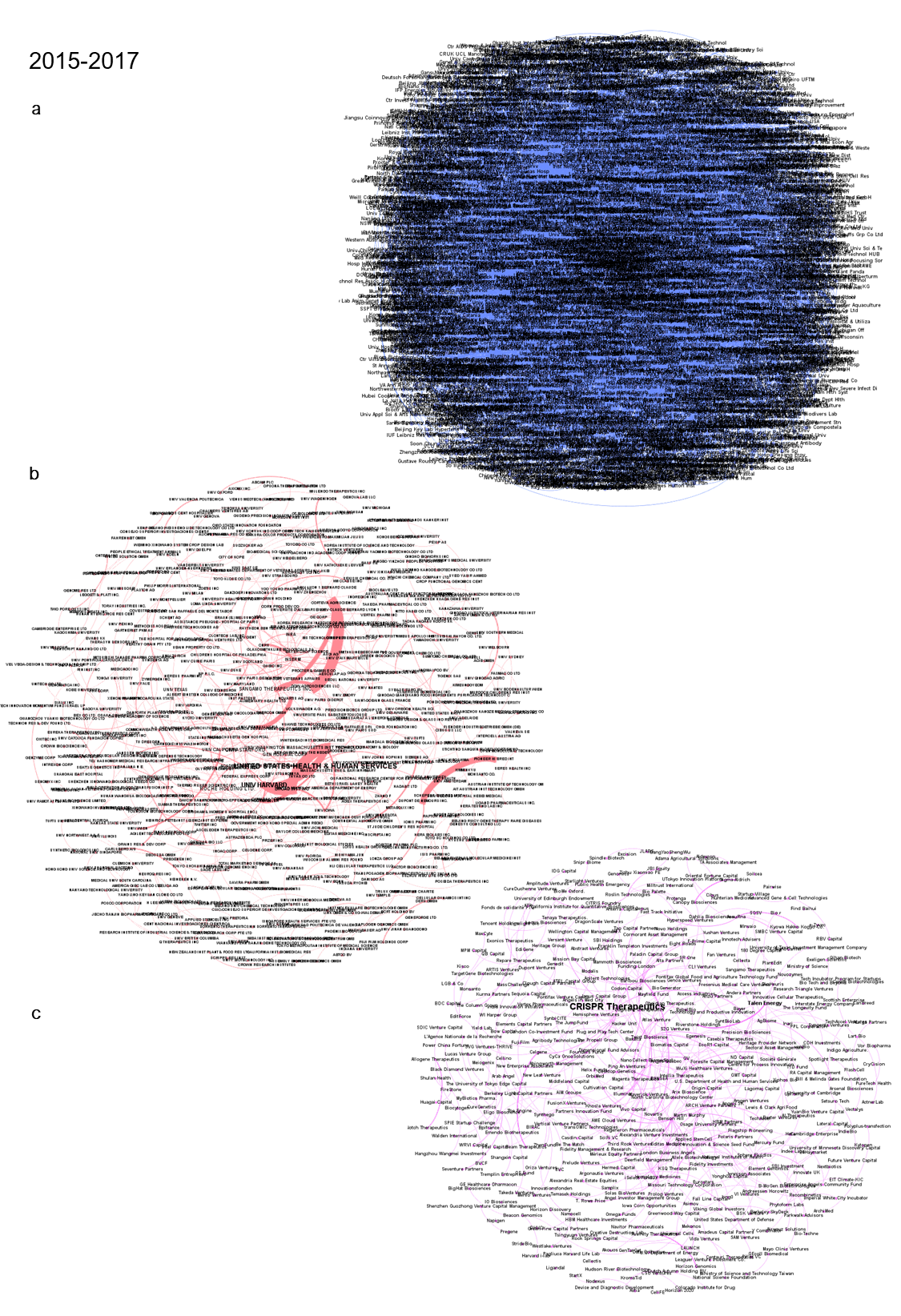
 **Supplementary Figure 8:** Networks generated from a publication (WoS) c patent (DII) and c company (Pitchbook) data, in the period from 2015-2017 visualized by Gephi.


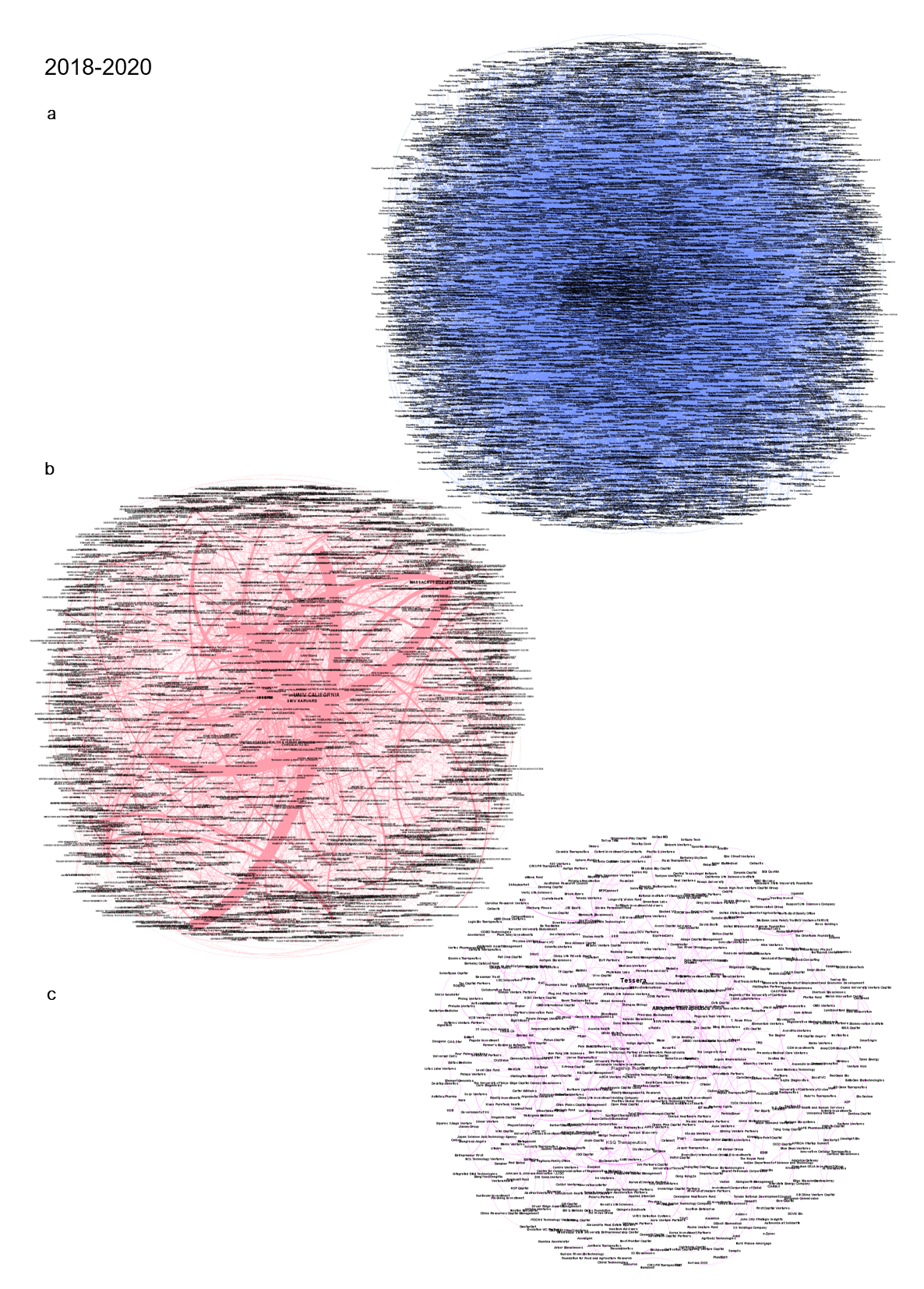


**Supplementary Figure 9:** Networks generated from a publication (WoS) c patent (DII) and c company (Pitchbook) data, in the period from 2018-2020, visualized by Gephi.

**Supplementary Table S2:** Abbreviations for Figure 2 (main text)

| 2i3T | 2i3T |
| --- | --- |
| Eight Roads | 8Rd |
| Aarhus Univ | AarU |
| Abcam | Abcam |
| AgBiome | AgBio |
| AGENCY FOR SCIENCE TECHNOLOGY & RESEARCH | AgST |
| Agribody Technologies | AgTech |
| Ahngook Pharm | AhnP |
| AIRBUS DEFENCE AND SPACE GMBH DE | AIRBUS |
| AI Therapeutics | AITh |
| Aldevron | AldV |
| Allele Biotechnology | AllBi |
| Allogene Therapeutics | AlloT |
| Altravax. | Altra |
| AMERSHAM PLC | AMERS |
| Amgen Ventures | AmVen |
| Andera Partners | AndP |
| Astellas Pharma Inc | APh |
| Applied StemCell | AppSC |
| ARCH Venture Partners | ARCH |
| Arima Genomics | Arima |
| Artemis Pharmaceuticals | ArtP |
| Aspen Neuroscience | AspN |
| ASSISTANCE PUBLIQUE HOPITAUX PARIS | AsPuH |
| Atlas Venture | AtV |
| Baylor Coll Med | BaColM |
| BASELL POLYOLEFINE GMBH | BASELL |
| BASF SE | BASF |
| Bayer Crop Sci | BCS |
| Benson Hill Biosyst | BenH |
| Berkeley Lights | BerkL |
| Beam Therapeutics | BeTh |
| Beam Therapeutics | BeTh |
| BioPontis Alliance | BioPA |
| BOSCH GMBH | BOSCH |
| Broad Inst | BrInst |
| Caribou Biosciences | CaBio |
| Carnegie Inst Sci | CarSc |
| Cellecta Inc | Cel |
| Cellectis SA | CellSA |
| Chinese Acad Sci | ChAc |
| Chinese Acad Med Sci | ChAcM |
| CIBERER | CIBERER |
| Cibus | Cibus |
| CNRS | CNRS |
| Colorado State Univ | ColSU |
| Columbia Univ | ColuU |
| CORTEVA AGRISCIENCE | CortA |
| CRISPR Therapeutics | CriT |
| Cropsolution | Crop |
| CSIR | CSIR |
| CuraGen Corporation | Cura |
| DAIMLER AG | DAIM |
| DEGUSSA AG | DEGU |
| Desktop Genetics | Desk |
| Dana Farber Canc Inst | DFCInst |
| Drug Farm | Drug |
| DuPont Co Inc | Du P |
| Univ Duke | DukU |
| Editas Medicine | Editas |
| Edigene Inc | EGen |
| Egenesis | Eges |
| Emory Univ | EmoU |
| EUROPEAN AERONAUTIC DEFENCE & SPACE | EU AER |
| Excision | Exis |
| FRAUNHOFER GESELLSCHAFT | FHG |
| Flagship Pioneering Inc | FlPio |
| FOND IMAGINE | FonI |
| Fudan Univ | FudU |
| FUJITSU LIMITED | FUJI |
| FUNDACIO CENT REGULACIO GENOMICA | FunRG |
| GE HEALTHCARE LTD | GE |
| GENERAL ELECTRIC COMPANY | GE |
| GEN HOSPITAL CORP | GeHoC |
| Genus | Gen |
| Gilead Sciences | GilSc |
| GLAXOSMITHKLINE BIOLOGICALS SA | GLAXO |
| GENERAL MOTORS CORP | GMO |
| HAMMERSMITH IMANET LTD | HAMM |
| Harvard Univ | HarU |
| HEALTHCARE CO LTD | HEAL |
| Heidelberg Univ | HeiU |
| Howard Hughes Med Inst | HHMInst |
| Horizon Discovery Group PLC | HoDi |
| Horizon Ventures | HoVen |
| HP INC | HP |
| HYUNDAI ELECTRONICS IND CO LTD | HYU |
| IB USP | IB USP |
| INST CARLOS SLIM DE LA SALUD A C | ICaSal |
| Inst Genom Res | IGenR |
| IMAGING RES SOLUTIONS LTD | IMA |
| Inari | Inari |
| INFINEON TECHNOLOGIES AG | INFIN |
| INRA | INRA |
| INSERM | INSERM |
| Intellia Therapeutics | IntelT |
| Inst Pasteur | IPast |
| Inst Rech Dev | IRechD |
| JAPAN TOBACCO INC | JapT |
| JLABS | JLAB |
| Johns Hopkins Univ | JoHo U |
| JST | JST |
| Kansas State Univ | KanSU |
| KANSAI PAINT CO. LTD. | KaPaint |
| Kings Coll London | KCL |
| Khosla Ventures | KhVen |
| KromaTid | KromT |
| KromaTid | KromT |
| KSQ Therapeutics | KSQT |
| Katholieke Univ Leuven | KUL |
| KWS SAAT AG | KWS |
| Kyoto Univ | KyoU |
| Kyoto Prefectural Univ | KyPU |
| LAM Therapeutics | LamT |
| Limelight Bio | LBio |
| LMU Munich | LMU |
| LOARANT CO | LOAR |
| Massachusetts Gen Hosp | MaGH |
| Magenta Therapeutics | MagT |
| Mammoth Biosciences | MammB |
| MaxCyte | MaxCyte |
| Mayo Clinic | Mayo |
| MICRONAS GMBH | MCRO |
| Massachusetts Eye and Ear Infirmary | MEE |
| Hannover Med Sch MHH | MHH |
| MASSACHUSETTS INST OF TECHNOLOY | MIT |
| MITSUBISHI ELECTRIC CORP | MITSU |
| MOTOROLA INC | MOT |
| MAX-PLANCK GESELLSCHAFT | MPG |
| Mem Sloan Kettering Canc Ctr | MSKCCtr |
| Mem Sloan Kettering Canc Ctr | MSKCCtr |
| NanoCellect Biomedical | NanoC |
| NCI | NCI |
| Natl Inst Agrobiol Sci | NIAgS |
| Novartis | Nova |
| NTI Nederlands Talen Instituut | NTI |
| Natl Univ Singapore | NUSin |
| NEWVECTYS | nVect |
| OSRAM GMBH | Osram |
| Pivot Bio | PBio |
| Peking Univ | PekU |
| Penn State Univ | PenSU |
| Pfizer | Pfiz |
| PlantEdit | PlaEd |
| Polyplus-transfection | PolyT |
| Precis BioSci | PreB |
| Precision BioSciences | PreB |
| Pairwise | Pws |
| Qihan Biotech | Quih |
| Recombinetics | Recom |
| Repare Therapeutics | ReTh |
| RIKEN | RIKEN |
| Roche Diagnost | RocD |
| Roche Venture Fund | Roch |
| ROCHE HOLDING LTD. | Roche |
| Rockefeller Univ | RockU |
| Russian Acad Med Sci | RuAcS |
| Sana Biotechnology | SaBio |
| Samplix | Sampx |
| SANGAMO THERAPEUTICS INC | SanT |
| Specific Biologics Inc | SBio |
| SCRIPPS RES INST | Scripps |
| SHIKOKU DENRYOKU KK | Shiko |
| Shizuoka Univ | ShiU |
| SIEMENS HEALTHCARE GMBH DE | SIEM |
| SIEMENS AG | SIEM |
| Sigma Aldrich Res Biotech | Sigma |
| Spanish Natl Canc Res Ctr | SNCR |
| Sorbonne Univ | SorU |
| Sphere Fluidics | Spher |
| Stanford Univ | StaU |
| State Res Ctr Appl Microbiol | StTaM |
| Suedzucker AG | Suedz |
| Synthego | Synt |
| Tessera | Tess |
| Topotarget | TopoT |
| Tsinghua Univ | TshU |
| Twist Bioscience | Twist |
| Univ Anhui | UAnh |
| Univ Bordeaux | UBor |
| Univ California | UCa |
| Univ Calif Berkeley | UCaB |
| Univ Calif Davis | UCaD |
| Univ China Agricultural | UCAgr |
| University of Calefornia at Irvine | UCaI |
| Univ Cambridge | UCam |
| Univ Calif San Diego | UCaSD |
| Univ Calif San Francisco | UCaSF |
| UNIV CALIFORNIA | UCL |
| Univ Calif Los Angeles | UCLA |
| Univ Copenhagen | UCop |
| UNIV CURIE PARIS | UCurie |
| Univ Erlangen-Nuernberg | UEr-N |
| UNIV FLORIDA | UFlo |
| Univ Freiburg | UFrei |
| Univ Hiroshima | UHir |
| Univ Illinois | UIln |
| UNIV IOWA | UIow |
| Natl Univ Ireland Univ Coll Cork | UIre |
| Univ Koeln | UKoe |
| Univ Kyoto | UKyo |
| Univ Claude Bernard Lyon 1 | ULyon |
| Univ Maryland | UMar |
| Univ Massachussetts | UMas |
| Univ Michigan | UMich |
| Univ Minnesota | UMinn |
| Univ Montpellier | UMon |
| Univ Nantes | UNan |
| Univ Oklahoma | UOkla |
| Univ Osaka | UOsa |
| Univ Ohio State | UOSt |
| Univ Oxford | UOxf |
| Univ Paris | UPar |
| Univ Paris 11 | UPar11 |
| UNIV PARIS DESCARTES | UParD |
| Univ Paris Saclay | UParSac |
| Univ Pennsylvania | UPen |
| Univ Pittsburgh | UPitt |
| Univ Princeton | UPrin |
| UNITED STATES HEALTH & HUMAN SERVICES | US HH |
| Univ So Calif | USCal |
| Univ Scotland | UScot |
| United States Health & Human Services | USHH |
| UNITED STATES HEALTH & HUMAN SERVICES | USHH |
| UNIV TEXAS | UTex |
| Univ Tech Kaiserslautern | UTKai |
| Univ Tokyo | UTok |
| University of Toronto | UTor |
| Univ Toronto | UToro |
| Univ Utah | UUta |
| Univ Utah | UUtah |
| Washington Univ | UWas |
| Washington Univ | UWas |
| Univ Wuerzburg Maximilian Julius | UWub |
| Univ Zurich | UZur |
| Vor Biopharma | VBio |
| Vertex Pharmaceuticals | VerPh |
| Vor Biopharma | VorB |
| Wageningen Univ | WagU |
| WHITEHEAD INST BIOMEDICAL RES | WhiBio |
| Yale Univ | YUni |
| Zhejiang Univ | ZheU |
| B-MoGen Biotechnologies | B-MoG |
| Boston Childrens Hosp | BostCH |
| Boston Univ | BosU |
| Cincinnati Childrens Hosp | CinnCH |
| GEORGIA TECH RES CORP | GIT |
| Homology Medicines | HomoM |
| Kumamoto Coll | KumaC |
| Mekonos | Meko |
| National Institutes of Health | NIH |
| OSPEDALE SAN RAFFAELE SRL | OspSR |
| Talen Energy | Talen |
| Texas Children Hosp | TexCH |
| UNIV GEORGIA | UGeo |
| Univ Sydney | USyd |
| Wellcome Trust Sanger Inst | WellT |
